# Prevalence and Radiological Features of Thoracic Ossification of the Ligamentum Flavum in Korea—A Retrospective Comparative Cohort Study Using MRI †

**DOI:** 10.3390/jcm15030952

**Published:** 2026-01-24

**Authors:** Junghyun Oh, Seong-Hwan Moon, Hak-Sun Kim, Kyung-Soo Suk, Chang-Ho Kang, Si Young Park

**Affiliations:** 1 Department of Orthopaedic Surgery, Severance Hospital, College of Medicine, Yonsei University, Seoul 03722, Republic of Korea; jungoh94@gmail.com (J.O.); shmoon@yuhs.ac (S.-H.M.); haksunkim@yuhs.ac (H.-S.K.); sks111@yuhs.ac (K.-S.S.); 2 Department of Radiology, Anam Hospital, College of Medicine, Korea University, Seoul 02841, Republic of Korea; mallecot@gmail.com

**Keywords:** ligamentum flavum, ossification, magnetic resonance imaging, epidemiology

## Abstract

(1) **Purpose**: Thoracic ossification of the ligamentum flavum (OLF) is increasingly recognized in East Asian populations, but reliable estimates in clinical settings remain limited. This study aimed to determine the clinic-based, lower thoracic (T8–T12) MRI prevalence of OLF among patients undergoing lumbar spine MRI for low-back pain and to identify radiological features associated with OLF. (2) **Materials and Method**: A cohort of patients with lower back pain who underwent L-Spine MRI studies in a tertiary medical center from January 2008 to December 2009 was created. Patients with thoracic OLF were identified, and a twice-fold sex-and-age-matched control group of patients without OLF, was randomly extracted. Radiological features in two groups were compared. (3) **Results**: The lower thoracic prevalence of OLF was 2.7%, significantly increasing in patients aged ≥60 years. OLF was most frequently involved in level T10-T11 (43%), and 23 cases (36%) showed multiple-level involvement. OLF was strongly associated with localized degenerative changes at the affected level, including higher degree of degenerative disc change, disc height loss, and more osteophyte formations. (4) **Conclusions**: Thoracic OLF is not a rare condition in patients with lower back pain. Patients with thoracic OLF were more likely to show features of focal degenerative changes, such as disc degeneration, osteophyte formation, and disc height loss on the level of OLF. Therefore, if initial plain radiographs of patients with neurologic deficits show evidence of degenerative change in the lower thoracic spine, a higher index of suspicion for thoracic OLF should prompt further evaluation.

## 1. Introduction

Degenerative changes in the thoracic spine and associated myelopathy are relatively uncommon conditions, in comparison to those in the cervical and lumbar spines [1]. In addition to its rarity, thoracic myelopathy shares clinical similarities with lumbar spine disease, leading clinicians to overlook and misdiagnose the condition [2]. Delayed diagnosis of thoracic myelopathy, however, can lead to devastating results with profound neurological deficits [1,3].

With the advent of advanced imaging, ossification of ligamentum flavum (OLF) has been recognized as a major cause of thoracic myelopathy [3,4,5]. Ligamentum flavum is a spinal ligament on the posterior column of the spine that provides stability [5]. Hypertrophy and ectopic endochondral ossification of the ligament in OLF by mechanical, endocrinal, and genetic factors cause spinal stenosis and myelopathy [6,7]. OLF mostly involves the thoracic spine—especially the more mobile lower thoracic region, where size progression and resultant myelopathy are common [8,9]. Although an undiagnosed OLF can potentially result in serious neurological compromise and delayed diagnosis of OLF is associated with poor surgical outcomes [10], there is little research on the epidemiological and radiological features of OLF. Few epidemiological investigations of OLF using X-rays, CT, and MRI have been published in East Asian countries, reporting inconsistent prevalence rates ranging from 3.8% to 71.8% in China, Korea, and Japan [11,12,13,14,15,16].

The aim of this study was to review both sagittal and axial cuts of magnetic resonance imaging (MRI) images in a tertiary medical center to estimate clinic-based prevalence of OLF in patients with lower back pain. Moreover, the objectives of this study were to compare radiologic features between patients with and without OLF and to identify imaging features that may serve as early diagnostic cues to this illusive yet potentially devastating condition.

## 2. Materials and Methods

### 2.1. Subjects

**Figure 1:**
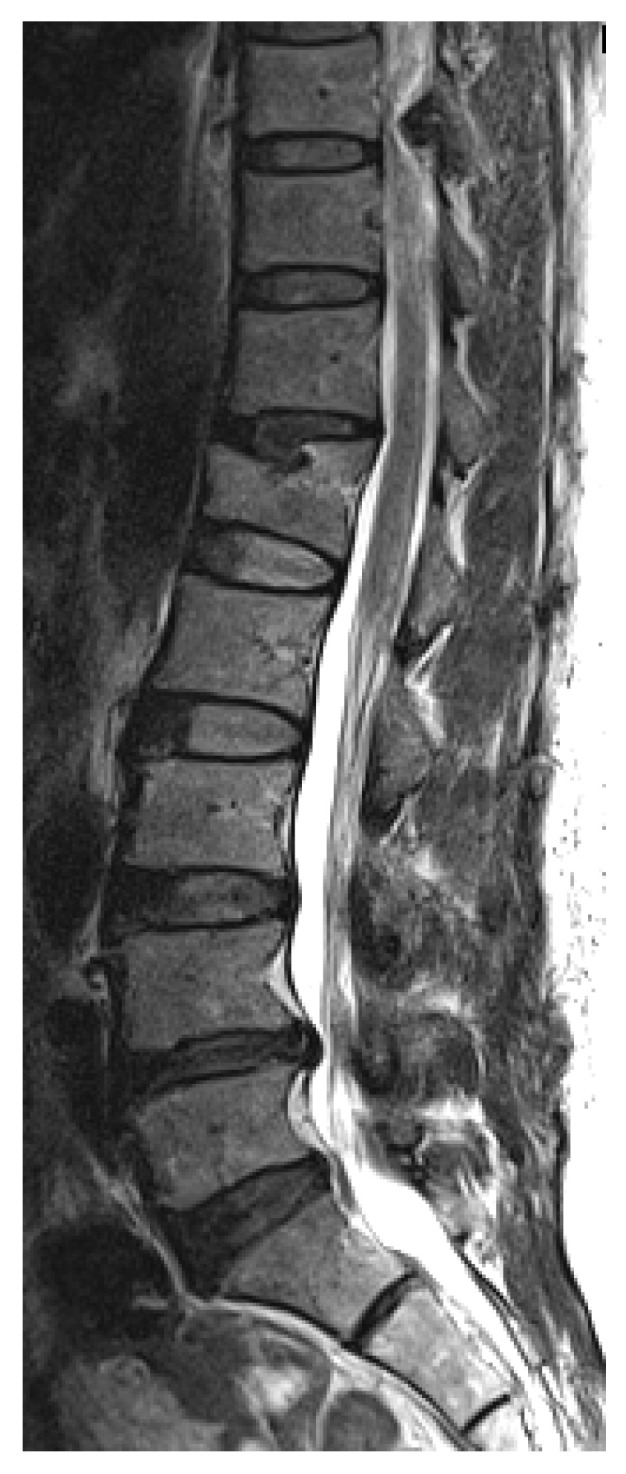
Representative MRI appearance of thoracic ossification of the ligamentum flavum (OLF). Sagittal T2-weighted image demonstrating focal low-signal ossification at the T10-11 level with associated dural indentation.

This study is a retrospective comparative cohort study that assessed all L-spine MRI image studies taken in a single tertiary referral center in South Korea between January 2008 and December 2009 as evaluation for lower back pain; 3.0-T MRI units (MAGNETOM Trio Tim; Siemens Healthcare, Erlangen, Germany) were used, which allowed for evaluation of the lower thoracic spine, including level up to T8. A cohort of 2480 patients (male: 1123 patients, female: 1357 patients) was formed with an age range of 8–94 years for male (mean: 51.6 ± 18.1 years) and 4–92 years for female (mean 58.5 ± 15.6 years). Sagittal and axial T2-weighted sequences of all MRI scans were independently reviewed by two radiologists specializing in musculoskeletal radiology with experience of more than five years in the subspecialty. In cases of disagreement, a third senior musculoskeletal radiologist reviewed the images, and a consensus decision was reached. OLF was identified as low-signal lesions on the posterior part of spinal canal on paramedian T2 sagittal images (Figure 1). Patients with ankylosing spondylitis or diffuse idiopathic skeletal hyperostosis were excluded using medical records. Plain radiographs and MRI cuts of each patient were reviewed for image findings of degenerative changes—degree of intradiscal degenerative change, the presence of osteophytes, presence of disc height loss, comorbid lumbar disease, and the presence of thoracolumbar kyphosis. This retrospective study was approved by the Institutional Review Board of Korea University Anam Hospital (IRB No. ED11309/K121301, approval date 9 March 2011). Given its retrospective design and use of anonymized data, the need for informed consent was waived.

### 2.2. A Retrospective Comparative Study

**Figure 2:**
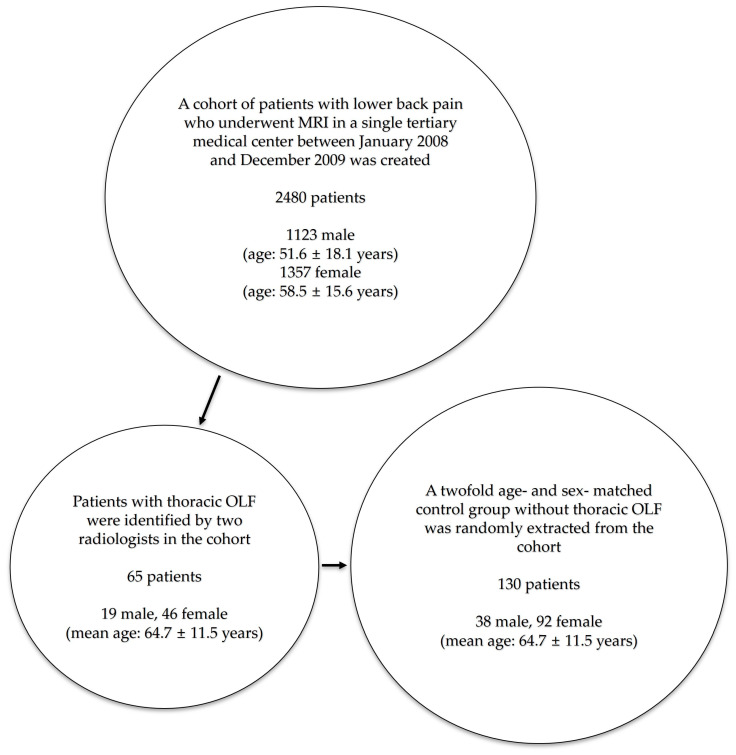
Study flow diagram illustrating the selection of 65 patients with thoracic OLF from a clinic-based MRI cohort of 2480 patients undergoing lumbar spine MRI for low-back pain (2008–2009), and the extraction of 130 age- and sex-matched controls (1:2 ratio) from the same cohort.

After identification of 65 patients with thoracic OLF, control subjects were selected from the same MRI cohort. For each OLF patient, two control patients without OLF were randomly selected from eligible candidates who were matched on age and sex. The case group of patients with OLF and the control group without OLF were compared based on their radiological features, observed via MRI and X-rays (Figure 2). Radiologists who evaluated imaging features were blinded to the case or control status at the time of image assessment.

### 2.3. Radiological Features

The formation of osteophytes, disc height loss, kyphotic change on the level affected with OLF were assessed on plain radiographs. Osteophyte formation was defined as the presence of anterior marginal bony outgrowths at the vertebral body endplates adjacent to the intervertebral disc space. Disc height loss was qualitatively assessed as an apparent reduction in disc height relative to the adjacent levels. These radiographic features were recorded as binary variables (present or absent). The extent of degenerative disc change shown in MRI was classified into five stages using the Pfirrmann method [17]. Such radiological features were compared between the case group and the control group.

### 2.4. Statistical Analysis

SPSS software (version 10.0; SPSS Inc., Chicago, IL, USA) was used for statistical analysis. Continuous variables are presented as means ± standard deviations after assessment of normality with the Kolmogorov–Smirnov test. Categorical variables are presented as counts or proportions and were compared using the chi-square test or Fisher’s exact test, as appropriate. For age-stratified prevalence of thoracic OLF and proportions of groups with degenerative radiological features, 95% confidence intervals (CIs) were estimated using the Wilson score method. Associations between thoracic OLF and radiological features were evaluated by calculating crude odds ratios (ORs) with 95% CIs. Multivariate logistic regression analyses were performed to obtain adjusted ORs. A two-sided *p*-value of less than 0.05 was considered statistically significant.

## 3. Results

### 3.1. Characteristics of Patients with OLF

**Figure 3:**
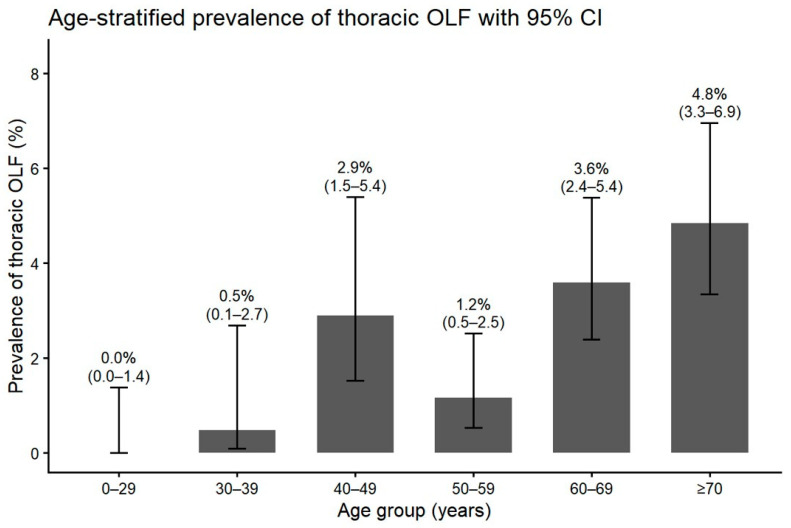
Age-stratified prevalence of thoracic OLF in the clinic-based MRI cohort (*n* = 2480). Prevalence (%) and 95% confidence intervals are shown for each age stratum (0–29, 30–39, 40–49, 50–59, 60–69, ≥70 years).

**Figure 4:**
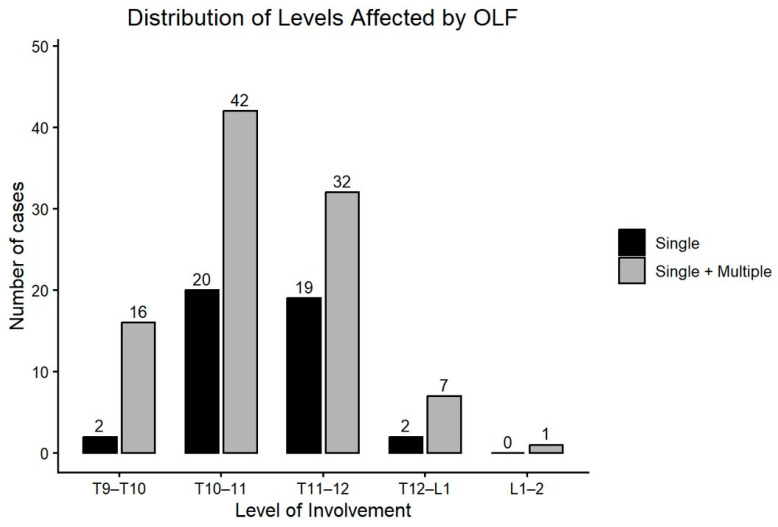
Distribution of OLF among 98 affected spinal levels in 65 patients, limited to lower thoracic levels (T8–T12) due to MRI coverage. T10–11 was the most involved level (43%).

Among 2480 patients included in this study, 1123 were male and 1357 were female; 65 patients showed image findings of thoracic OLF, indicating a prevalence of 2.6%. Among the 65 patients, 19 patients were male and 46 were female, showing OLF is more common in females than males (Fisher Exact test; *p* = 0.008). The average age of the case group was 64.7 ± 11.5 years. Older age than 60 showed higher incidence of OLF, which was statistically significant (X^2^-test; *p* < 0.001) (Figure 3). A total of 13 cases of OLF (19%) showed symptoms of thoracic myelopathy; 43 cases (65%) showed involvement of OLF at a single level, while 23 cases (35%) had OLF at multiple levels. Among a total of 98 levels with OLF, the most frequently involved level with OLF was the T10 to T11 level (43%). The level with the second highest incidence was the T11 to T12 level (33%), and the third was the T9 to T10 level (17%) (Figure 4).

### 3.2. Comparison Between the OLF Group and the Non-OLF Group

A total of 130 age- and sex-matched control patients without thoracic OLF were randomly selected from the same MRI cohort in a 1:2 ratio. The control group consisted of 38 males and 92 females, with a mean age of 64.7 ± 11.5 years, equal to that of the OLF group. There was no significant difference between the OLF and control groups in the presence of comorbid lumbar disease or thoracolumbar kyphosis (Table 1).

**Figure 5:**
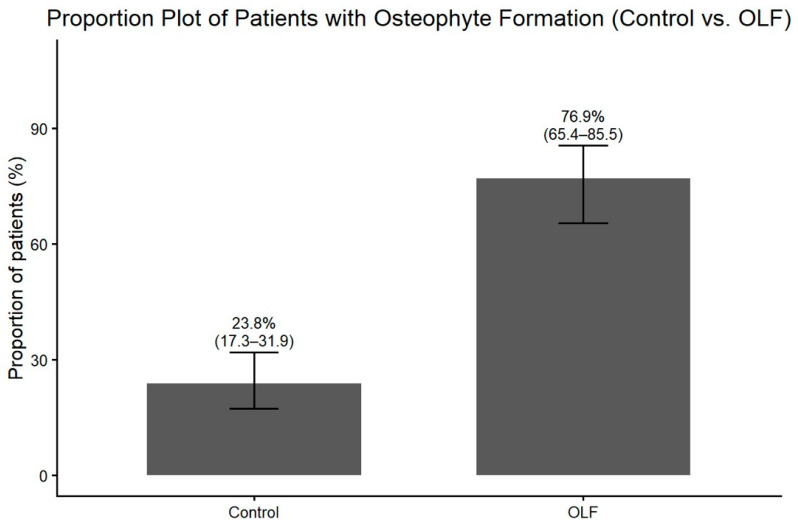
Proportion (%) of patients with anterior osteophyte formation at the OLF-involved level in the OLF group (*n* = 65) versus age- and sex-matched controls (*n* = 130), with 95% confidence intervals.

**Figure 6:**
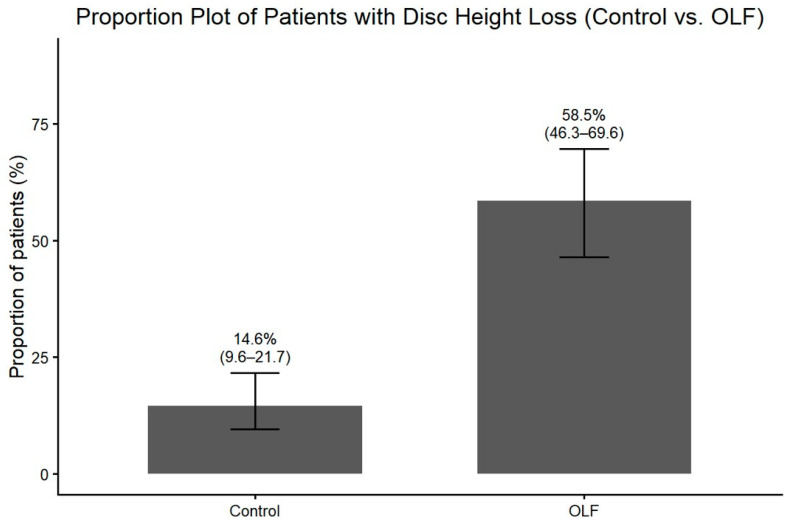
Proportion (%) of patients showing disc height loss at the OLF-involved level in the OLF group (*n* = 65) versus matched controls (*n* = 130), with 95% confidence intervals.

**Figure 7:**
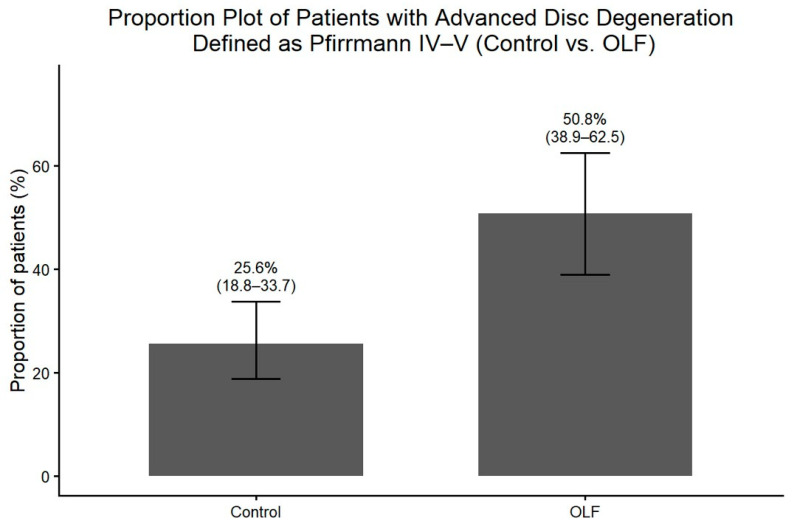
Distribution of intervertebral disc degeneration grades (Pfirrmann I–V) at the OLF-involved level in OLF patients (*n* = 65) and matched controls (*n* = 130). Advanced degeneration (grades IV–V) was significantly more common in the OLF group.

Radiographic assessment revealed that degenerative changes at the level affected by OLF were significantly more prevalent in the OLF group. Osteophyte formation on plain radiographs was observed more frequently in OLF group than in the control group (Figure 5). Similarly, disc height loss at the affected level was more prevalent in patients with OLF (Figure 6). These associations between OLF and degenerative radiographic features remained significant after adjustment for comorbid lumbar disease and thoracolumbar kyphosis (Table 2). MRI-based assessment demonstrated that the degree of intervertebral disc degeneration, evaluated using the Pfirrmann grading system, was significantly higher in the OLF group than in the control group. When advanced disc degeneration (Pfirrmann grade IV–V) was analyzed as a binary outcome, patients with OLF exhibited a significantly higher prevalence compared to controls (crude OR, 3.00; 95% CI, 1.60–5.62), which persisted after adjustment (adjusted OR, 3.44; 95% CI, 1.74–6.79) (Figure 7).

## 4. Discussion

This MRI cohort study revealed that the clinic-based prevalence of OLF involving the lower thoracic spine (T8–T12) is 2.6% in patients with lower back pain, which opposes the traditionally held belief that OLF is a rare condition. The incidence is even higher in patients over age 60, and the result of our study echoes findings of recent studies that OLF is a common finding among the aging Asian population [5].

Previous epidemiological studies, all of which were carried out in East Asian countries, have reported a wide range of incidence for OLF [5]. Population studies in Japan have reported incidence of OLF between 12% and 36% [13,18]. Meanwhile, a Chinese study reviewing whole-spine MRI of 1700 volunteers reported the rate of OLF to be 3.8% [11]. A previous Korean population study by Moon et al. [14]. reviewed L-spine MRI of patients with back pain or leg pain—a similar study design to our study. The study estimated the prevalence of thoracic OLF to be 16.9%, which is substantially higher than our estimate. The difference likely resulted from different modes of diagnosis for OLF. While our study reviewed both axial and sagittal cuts of the lower thoracic spine, a study by Moon et al. based the diagnosis only on whole T2 sagittal cuts, which may have led to an overestimation of OLF, as differential diagnosis with ligamentum flavum hypertrophy or infolding on sagittal views can be difficult. Also, MRI coverage in our study was limited to the lower thoracic spine, so the true prevalence of thoracic OLF is likely underestimated, as OLF involving the upper and mid-thoracic levels was not assessed.

In contrast, a study using computed tomography (CT), which is considered more sensitive and specific for detecting ossified lesions, reported incidental thoracic OLF rates exceeding 20% [15]. Although axial MRI improves diagnostic specificity compared with sagittal-only assessment, thin or early ossified OLF lesions may still be misclassified as hypertrophic ligamentum flavum. Such misclassification biased prevalence estimates toward under-detection, supporting the interpretation that the prevalence estimate by this study likely represents a lower-bound estimate of true thoracic OLF prevalence. A future study that incorporates both MRI and CT scans should yield a more precise incidence rate. As over 19% of patients with OLF showed clinical signs of myelopathy in our study, OLF should not be overlooked as an uncommon, inconsequential condition, and clinicians should consider OLF as a possible etiology of myelopathy, especially in the Asian population.

Radiographic analysis demonstrated that OLF was strongly associated with localized degenerative change at the affected spinal level—disc degeneration, osteophyte formation, and disc height loss. On the other hand, coexistent lumbar spine pathology and thoracolumbar kyphosis were not statistically different between OLF and control groups. These findings that OLF is associated with degenerative spondylosis only at the level of OLF suggest a localized biomechanical etiology of OLF. Recent studies explain OLF as a result of focal mechanical stress and microtrauma, which cause hypertrophy and trigger subsequent ossification of the ligament [19,20]. Repeated stress may have caused disc degeneration, disc height loss, and osteophyte formation, as well as the formation of OLF. The highest incidence of OLF in the T10 to T11 level—where free ribs in the lower thoracic spine allow for more motion and susceptibility to biomechanical stress—also support the view on OLF a result of wear and tear. Older age was associated with higher prevalence of OLF—consistent with previous research—and such increasing incidence of OLF with older age supports the notion that OLF is a manifestation of a degenerative cascade of genetically prone individuals [21,22,23].

Thoracic myelopathy caused by OLF presents with an insidious onset of myelopathic symptoms—patients commonly complain of gait disturbance, motor weakness, and non-dermatomal sensory change of lower extremity [2]. Thoracic myelopathy by OLF presents a diagnostic challenge, as it presents with ominous clinical onset and shares clinical similarities with lumbar spinal disease [1]. Delays in diagnosis can be potentially devastating, as a longer duration of preoperative symptoms is a significant prognostic factor associated with poor surgical outcomes [1]. As this study shows, thoracic OLF is highly associated with degenerative spondylosis of the level, so degenerative changes in initial plain radiographs of the thoracic spine, particularly in older patients, should trigger a higher index of suspicion for thoracic OLF as a cause of thoracic myelopathy. While CT combined with MRI remains the diagnostic gold standard for OLF, selective use of these modalities based on radiographic and clinical cues may help balance diagnostic accuracy with cost and radiation exposure. Prompt CT and MRI should be carried out not only for the lumbar region but also for the thoracic region, if patients with myelopathic symptoms show degenerative changes in the thoracic spine.

This study has several limitations. First, the cohort consisted of patients undergoing lumbar spine MRI for lower back pain at a single tertiary referral center, introducing selection bias and limiting generalizability to the general population. Secondly, MRI coverage was restricted to the lower thoracic spine, which resulted in underestimation of overall thoracic OLF prevalence. Furthermore, OLF was defined based on MRI without CT confirmation. Early or thin ossified lesions may have been misclassified as hypertrophic ligamentum flavum, potentially leading to under-detection of OLF. Taken together, because MRI alone is less sensitive than CT for detecting thin or early ossified lesions, the degree of underestimation in our study is likely non-trivial and affects both the observed prevalence and the strength of association with degenerative changes by shifting borderline cases into the non-OLF category. Also, this study was not a fully randomized controlled trial. Still, all patients undergoing an MRI scan for lower back pain in a single tertiary medical center were collected in this study’s cohort, which provided a large, heterogeneous group of patients. We believe the two-fold age-matched, sex-matched randomly extracted control group minimized major confounding variables and provided a meaningful comparison to find distinct radiological features of OLF. Although diabetes mellitus has been discussed as a potential risk factor for OLF in the prior literature, BMI and DM variables were not available in analyzable form in this dataset and were therefore excluded from multivariable modeling. Accordingly, our adjusted ORs should be interpreted as adjusted for spine-related covariates only. Future research should focus on prospective, multi-center investigations with broader imaging protocols (including CT) and biomechanical analysis to better elucidate the pathogenesis of OLF. Genetic or metabolic profiling of affected individuals may also yield important insights into susceptibility factors.

This study is the first to report radiological features that are statistically significantly associated with OLF. Such findings—disc height change, osteophyte formation—are readily identifiable on plain radiographs and can serve as useful diagnostic cues for OLF. The golden standard of diagnosis for OLF is a combination of a CT scan and MRI [5,24]. As routinely using these image modalities poses both a radiological hazard and financial burden for patients, early identification of patients with high propensity for OLF is important, so that a select number of patients can undergo the necessary costly evaluation. Our study suggests focal degenerative change on the thoracic spine should raise a higher index of suspicion for OLF. Also, clinicians should keep in mind that older age is a risk factor for OLF. In the context of the rapidly aging Asian population, the incidence of thoracic OLF and the resultant thoracic myelopathy should increase with time. It is important that clinicians should not overlook thoracic OLF as a rare condition. This study provides helpful diagnostic clues that should prompt further investigation and early intervention that can potentially prevent devastating neurologic consequences of a misdiagnosed, undiagnosed thoracic myelopathy.

## 5. Conclusions

This clinic-based cohort study analyzing MRI with partial thoracic coverage demonstrates that OLF is not a rare incidental finding but a relatively common condition, particularly among elderly Korean patients undergoing evaluation for low-back pain. Our findings suggest that OLF may be a focal manifestation of chronic mechanical stress and degenerative spondylosis, rather than a purely systemic process. Importantly, OLF was significantly associated with localized degenerative changes at the spinal levels involved, including intervertebral disc degeneration, disc height loss, and osteophyte formation. These radiologic markers may serve as early diagnostic cues in clinical practice. Clinicians should maintain a high index of suspicion for thoracic OLF in elderly patients presenting with myelopathic symptoms, especially when degenerative changes are visible on thoracic spine imaging.

## Figures and Tables

**Table 1:** Baseline characteristics of patients with thoracic OLF (*n* = 65) and age- and sex-matched controls without OLF (*n* = 130), drawn from a clinic-based MRI cohort of 2480 patients undergoing lumbar spine MRI for low-back pain.

Characteristics	OLF Group (*n* = 65)	Control Group (*n* = 130)	*p*-Value
Age (mean ± SD, years)	64.7 ± 11.5	64.7 ± 11.5	matched
Sex, *n* (%)			matched
Male	19 (29.2%)	38 (29.2%)	
Female	46 (70.8%)	92 (70.8%)	
Presence of Thoracolumbar Kyphosis, *n* (%)	3 (4.6%)	16 (12.3%)	0.15
Comorbid Lumbar Disease, *n* (%)			0.12
Herniated Nucleus Pulposus	15 (23.1%)	43 (33.1%)	
Spinal Stenosis	50 (76.9%)	87 (66.9%)	

**Table 2:** Crude and adjusted odds ratios (ORs) for the associations between thoracic OLF and radiological features at the affected spinal level, including osteophyte formation, disc height loss, and advanced disc degeneration (Pfirrmann IV–V). Adjusted ORs control for comorbid lumbar disease and thoracolumbar kyphosis.

Radiological Features	Crude OR (95% CI)	*p*-Value	Adjusted OR (95% CI)	*p*-Value
Osteophyte formation	10.65 (5.27–21.52)	<0.001	13.87 (6.38–30.16)	<0.001
Disc height loss	8.22 (4.11–16.44)	<0.001	10.24 (4.78–21.95)	<0.001
Advanced disc degeneration (Pfirrmann IV–V)	3.00 (1.60–5.62)	<0.001	3.44 (1.74–6.79)	<0.001

## Data Availability

The data presented in this study are available on request from the corresponding author. The data are not publicly available due to privacy or ethical restrictions.

## References

[1] Shiokawa K., Hanakita J., Suwa H., Saiki M., Oda M., Kajiwara M. (2001). Clinical analysis and prognostic study of ossified ligamentum flavum of the thoracic spine. J. Neurosurg..

[2] Ben Hamouda K., Jemel H., Haouet S., Khaldi M. (2003). Thoracic myelopathy caused by ossification of the ligamentum flavum: A report of 18 cases. J. Neurosurg..

[3] Fan T., Sun C., Chen G., Jiang S., Li W., Chen Z. (2023). Clinical progression of ossification of the ligamentum flavum in thoracic spine: A 10- to 11-year follow-up study. Eur. Spine J..

[4] Sato T., Kokubun S., Tanaka Y., Ishii Y. (1998). Thoracic myelopathy in the Japanese: Epidemiological and clinical observations on the cases in Miyagi Prefecture. Tohoku J. Exp. Med..

[5] Daniels A.H., McDonald C.L., Basques B.A., Kuris E.O. (2022). Ossified Ligamentum Flavum: Epidemiology, Treatment, and Outcomes. J. Am. Acad. Orthop. Surg..

[6] Zhang C., Chang Y., Shu L., Chen Z. (2024). Pathogenesis of thoracic ossification of the ligamentum flavum. Front. Pharmacol..

[7] Nishikawa M., Yoshimura M., Naito K., Yamagata T., Goto H., Hara M., Ikuno H., Goto T. (2024). The Symptomatic Calcification and Ossification of the Ligamentum Flavum in the Spine: Our Experience and Review of the Literature. J. Clin. Med..

[8] Aizawa T., Sato T., Tanaka Y., Ozawa H., Hoshikawa T., Ishii Y., Morozumi N., Ishibashi K., Kasama F., Hyodo H. (2006). Thoracic myelopathy in Japan: Epidemiological retrospective study in Miyagi Prefecture during 15 years. Tohoku J. Exp. Med..

[9] Tang C.Y.K., Cheung K.M.C., Samartzis D., Cheung J.P.Y. (2021). The Natural History of Ossification of Yellow Ligament of the Thoracic Spine on MRI: A Population-Based Cohort Study. Glob. Spine J..

[10] Ramesh Chandra V.V., Prasad B.C.M., Rajesh P., Agarwal S., Krishna M.M. (2022). Factors Predicting Poor Surgical Outcome in Patients with Thoracic Ossified Ligamentum Flavum—Analysis of 106 Patients in a Tertiary Care Hospital in South India. Neurol. India.

[11] Guo J.J., Luk K.D., Karppinen J., Yang H., Cheung K.M. (2010). Prevalence, distribution, and morphology of ossification of the ligamentum flavum: A population study of one thousand seven hundred thirty-six magnetic resonance imaging scans. Spine.

[12] Lang N., Yuan H.S., Wang H.L., Liao J., Li M., Guo F.X., Shi S., Chen Z.Q. (2013). Epidemiological survey of ossification of the ligamentum flavum in thoracic spine: CT imaging observation of 993 cases. Eur. Spine J..

[13] Mori K., Kasahara T., Mimura T., Nishizawa K., Murakami Y., Matsusue Y., Imai S. (2013). Prevalence, distribution, and morphology of thoracic ossification of the yellow ligament in Japanese: Results of CT-based cross-sectional study. Spine.

[14] Moon B.J., Kuh S.U., Kim S., Kim K.S., Cho Y.E., Chin D.K. (2015). Prevalence, Distribution, and Significance of Incidental Thoracic Ossification of the Ligamentum Flavum in Korean Patients with Back or Leg Pain: MR-Based Cross Sectional Study. J. Korean Neurosurg. Soc..

[15] Kim S.-I., Ha K.-Y., Lee J.-W., Kim Y.-H. (2018). Prevalence and related clinical factors of thoracic ossification of the ligamentum flavum-a computed tomography-based cross-sectional study. Spine J..

[16] Choi Y.H., Lee J.H., Kwon Y.M. (2022). Distribution, and Concomitance of Whole-Spine Ossification of the Posterior Longitudinal Ligament and Ossification of the Ligament Flavum in South Koreans: A Whole-Spine-CT-Based Cross-Sectional Study. Neurospine.

[17] Pfirrmann C.W.A., Metzdorf A., Zanetti M., Hodler J., Boos N. (2001). Magnetic resonance classification of lumbar intervertebral disc degeneration. Spine.

[18] Fujimori T., Watabe T., Iwamoto Y., Hamada S., Iwasaki M., Oda T. (2016). Prevalence, Concomitance, and Distribution of Ossification of the Spinal Ligaments: Results of Whole Spine CT Scans in 1500 Japanese Patients. Spine.

[19] Kaneyama S., Doita M., Nishida K., Shimomura T., Maeno K., Tamura Y., Kurosaka M., Yonenobu K. (2008). Thoracic myelopathy due to ossification of the yellow ligament in young baseball pitchers. J. Spinal Disord. Tech..

[20] Yoshiiwa T., Miyazaki M., Kawano M., Ikeda S., Tsumura H. (2016). Analysis of the Relationship Between Hypertrophy of the Ligamentum Flavum and Lumbar Segmental Motion with Aging Process. Asian Spine J..

[21] Ye Z., Yin X., Ao J., Qin J., Wei Z., Qian H. (2025). Biological mechanisms underlying the ossification of ligamentum flavum and potential therapeutic targets derived from current research. Eur. Spine J..

[22] Zhang H., Deng N., Zhang L., Zhang L., Wang C. (2022). Clinical Risk Factors for Thoracic Ossification of the Ligamentum Flavum: A Cross-Sectional Study Based on Spinal Thoracic Three-Dimensional Computerized Tomography. Risk Manag. Healthc. Policy.

[23] Yamada T., Shindo S., Yoshii T., Ushio S., Kusano K., Miyake N., Arai Y., Otani K., Okawa A., Nakai O. (2021). Surgical outcomes of the thoracic ossification of ligamentum flavum: A retrospective analysis of 61 cases. BMC Musculoskelet. Disord..

[24] Hirabayashi S. (2017). Ossification of the ligamentum flavum. Spine Surg. Relat. Res..

